# Suture-Mediated Delivery System Reduces the Incidence of Uterine Scarring Through the TGF-β Pathway

**DOI:** 10.3390/jfb16020052

**Published:** 2025-02-07

**Authors:** He Bai, Wei Zhang, Xuanxuan Yan, Lin Qiu, Pengfei Cui, Weiyang Chen

**Affiliations:** 1 School of Pharmacy, Changzhou University, Changzhou 213164, China; s22091055029@smail.cczu.edu.cn (H.B.); 2200790116@smail.cczu.edu.cn (W.Z.); 2200790115@smail.cczu.edu.cn (X.Y.); linqiu@cczu.edu.cn (L.Q.); 2 Changzhou Maternity and Child Health Care Hospital, Changzhou 213003, China

**Keywords:** uterine scar, suture, RhCol III, gene delivery, TGF-β3

## Abstract

In recent years, factors such as the postponement of childbearing and the relaxation of the childbearing policy have led to an increase in the proportion of cesarean sections and other intrauterine surgeries among pregnant women, further increasing the incidence of uterine scars. Currently, there is a lack of effective clinical treatment methods for uterine scars. In this study, a suture loaded with gene medicine was designed for the repair of uterine scars. Specifically, the non-viral vector Lipo8000 was first used to form a complex solution with the plasmid TGF-β3. Then, it was mixed and adsorbed with the surgical sutures pretreated with recombinant human type III collagen (RhCol III). In vitro experiments confirmed that RhCol III and the plasmid were successfully loaded onto the sutures and could be released and expressed. In vivo experiments were carried out using a rat model simulating uterine scars. The section results showed that compared with the scar model group, the expression level of TGF-β3 in the RhCol III+TGF-β3 group increased by 39%, the expression level of TGF-β1 decreased by 62.8%, and the fibrosis rate decreased by 16.8%, which has a positive effect on the prevention of uterine scars. This study integrates the therapeutic medicine into the sutures, ensuring that the medicine can come into contact with the wound site after suturing. Moreover, RhCol III and the gene medicine work synergistically to promote the repair of uterine wounds.

## 1. Introduction

Uterine scar typically refers to localized impairment of blood supply at the surgical site, impacting cellular repair and tissue regeneration processes at the incision site, subsequently to surgeries such as cesarean section (CS) and hysterectomy for uterine fibroids, due to trauma inflicted on the uterine wall. As the wound heals, scar tissue gradually forms and replaces the original muscle tissue [1]. CS, as a clinical intervention, is utilized to manage high-risk pregnancies and dystocia, and its judicious use can effectively reduce mortality rates among high-risk pregnant women and perinatals. However, due to various factors such as economic and social development and delayed childbearing age, the cesarean section rate in China has remained high in recent years, exceeding 40%, far surpassing the 15% recommended by the World Health Organization [2,3,4]. The production of scar and the accumulation of fibrotic connective tissue during the healing process of uterine wounds readily lead to the formation of cesarean scar diverticulum (CSD). The presence of CSD disrupts the normal shedding and repair processes of the endometrium, affecting fertilization and implantation, thereby posing multiple associated risks [5]. Firstly, it may cause prolonged menstrual bleeding and increased menstrual flow, causing inconvenience to women’s lives. Secondly, it may impair fertilization and implantation, leading to infertility and habitual abortions. Furthermore, scarred uterus pregnancies may result in severe complications, even threatening the lives of both mothers and infants [6]. The presence of uterine scars may also trigger complications such as chronic abdominal pain and pelvic inflammatory diseases. These adverse effects not only impact reproductive health but may also adversely affect quality of life and mental health [7]. Therefore, it is particularly important to promptly identify the etiology of uterine scar formation and select appropriate treatment methods.

The etiology of uterine scar formation may be categorized into two aspects. From a macro perspective, the number of cesarean sections and suturing techniques can influence scar formation. Age is also a significant factor, as older parturients often have complications such as obesity and gestational diabetes, and recovery becomes more difficult with advancing age [8]. On the other hand, from a micro perspective, scar formation involves excessive deposition of extracellular matrix proteins, particularly fibrin. Normally, the uterine myometrium is composed primarily of smooth muscle tissue and a small amount of collagen fibers in a ratio close to 7:2. The healing of uterine incisions after cesarean section is a complex biological process involving a series of inflammatory responses, granulation tissue proliferation, tissue repair, and remodeling [9]. Although a small number of smooth muscle cells can regenerate after cesarean section, the primary repair mechanism is through granulation tissue proliferation, with scar formation filling the defect to maintain the structural integrity of tissues and organs. Over time, scar tissue may exhibit excessive proliferation and fibrosis, altering the tensile strength and morphology of the scar site. The tissue cells at the scar site can secrete various growth factors, which not only stimulate excessive proliferation of granulation tissue but also promote the synthesis and secretion of extracellular matrix components such as collagen and fibronectin [10]. This hinders the uterus from recovering its original functional state and induces various secondary reactions.

Currently, clinical treatments for uterine scars after cesarean section primarily include hormonal therapy and surgical intervention. Among them, non-surgical treatment is the preferred method for poor healing of uterine incisions after cesarean section. Hormonal therapy, represented by pharmacological treatments, can alleviate symptoms to a certain extent, such as shortening the menstrual cycle to gradually restore normalcy, reducing bleeding volume, and mitigating and preventing inflammation [11]. However, its application is limited due to adverse reactions associated with long-term medication, poor patient compliance, and possible recurrence after discontinuation [12]. If non-surgical treatment is ineffective or the condition worsens, surgical treatment should be considered. Surgical options include hysteroscopic electrocautery, hysteroscopic repair, transvaginal scar excision and suturing, laparoscopic surgery, and laparotomy for excision and suturing [13]. These treatment methods are more often used as remedial measures and do not address the underlying problem, especially surgical interventions, which not only require high surgical skills but may also cause secondary harm to patients [14,15]. Therefore, finding simpler and more effective treatment methods or reducing their incidence is an important research direction.

Gene therapy refers to the introduction of normal or therapeutic genes into target cells through molecular biological methods to correct or compensate for diseases caused by genetic defects and abnormalities, thereby achieving the effect of treating or improving specific diseases. Over the past few decades, more than 30 gene therapy medicine have been marketed, playing a crucial role in fields such as cancer, congenital genetic diseases, and vaccine immunology [16]. Additionally, research on gene therapy for the treatment of uterus-related diseases has been conducted and has achieved certain results [17,18]. However, due to the special location of uterine diseases and the inconvenience of medicine administration, this field still lacks extensive research.

From the micro-mechanism of the pathogenesis of uterine scars, the transforming growth factor-β (TGF-β) family is one of the main components of the extracellular matrix and is closely related to scar proliferation [19,20,21,22]. They bind to specific receptors on the cell surface and activate specific promoters through specific signaling pathways, initiating the expression of related genes [23,24]. Their primary role is to promote the synthesis of fibroblasts, causing them to produce collagen and extracellular matrix components, thereby enhancing the tensile strength of wounds [25,26]. During the wound healing process, the expression of their receptors increases, leading to increased fibrotic activity. The excessive deposition of collagen fibers or overexpression of fibroblasts can result in excessive fibrosis of scars [27]. TGF-β1 is one of the most potent profibrotic factors in the TGF-β family, and its overexpression can lead to excessive scar proliferation, fibrosis, and aging [28]. On the other hand, embryonic wound microenvironments have higher levels of TGF-β3 and lower levels of TGF-β1. Furthermore, the addition of exogenous TGF-β3 to wounds can promote scarless healing. This scarless healing may be due to the higher expression of TGF-β3 relative to TGF-β1 [29,30].

Additionally, Type III collagen is a naturally occurring collagen type in human tissues such as skin, fascia, and tendons. It is primarily synthesized through natural pathways within the human body and is abundant in infant skin, serving as a crucial component that supports skin tenderness and fineness. As age increases, its content in the skin gradually decreases, contributing to skin aging [31]. RhCol III, however, is produced through modern biotechnology such as gene recombination and biofermentation, based on the original gene sequence of human skin Type III collagen. Its production process does not involve direct extraction from human bodies and avoids the risk of contamination by animal-derived pathogens [32,33,34]. With advancements in synthetic biology, it has garnered increasing attention in the biomedical field and demonstrated significant application potential in wound healing. By promoting the growth of granulation tissue at wound sites, it subsequently facilitates cell migration, proliferation, and differentiation. These biological effects contribute to accelerating the wound healing process and shortening treatment duration [35,36,37]. Therefore, the exogenous supplementation of RhCol III aids in forming a temporary extracellular matrix, inducing directed migration of relevant cells, thereby regulating cell proliferation, migration, and differentiation. This has a positive impact on reducing scar tissue formation.

**Figure 1:**
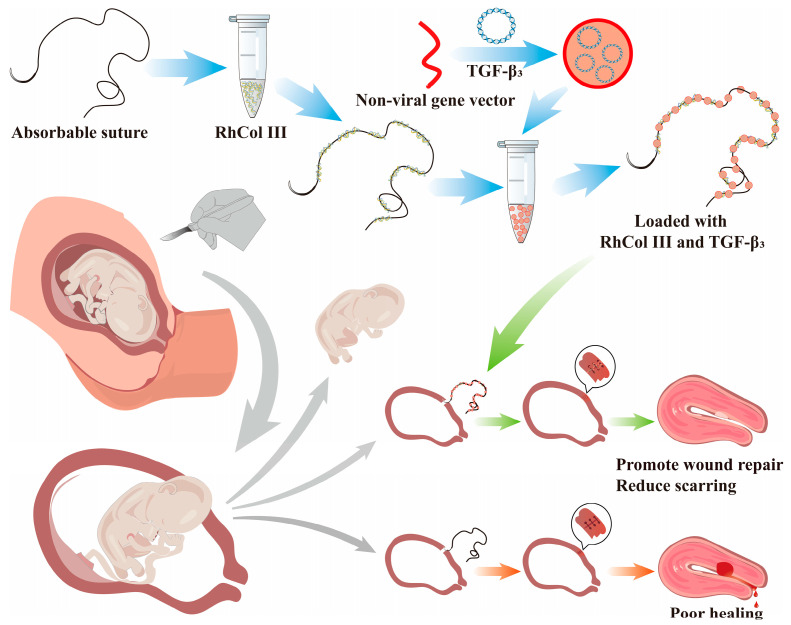
Schematic representation of the preparation method for sutures loaded with RhCol III and gene-based therapeutic TGF-β3, and the application in preventive treatment of uterine scarring.

Based on these principles, we have designed a suture-mediated gene medicine delivery system aimed at reducing the incidence of uterine scars. This system initially utilizes absorbable sutures combined with RhCol III (hereinafter referred to as RhCol III for simplicity), which not only aids in wound healing but also enhances the suture’s adsorption capacity. Subsequently, a highly biocompatible non-viral vector, Lipo8000, is used to form a composite solution with the therapeutic gene TGF-β3. This composite solution is mixed with surgical sutures coated with RhCol III, enabling the sutures to adsorb the gene medicine. During surgeries such as cesarean sections, these medicine-loaded sutures are used for wound closure. Postoperatively, the gene medicine gradually diffuses from the sutures to the wound site and enters adjacent cells through endocytosis to express the corresponding proteins. This process upregulates the ratio of TGF-β3 to TGF-β1, promoting wound healing and recovery while reducing fibrosis accumulation, ultimately aiming to decrease the incidence of scars and tissue fibrosis. The method designed in this study is simple and feasible, achieving direct medicine delivery to the lesion site through suture-based medicine loading. It provides a novel therapeutic approach for uterine scars caused by inflammatory responses in surgeries such as cesarean sections (Figure 1).

## 2. Materials and Methods

### 2.1. Materials

The absorbable surgical suture PGA (Shanghai Pudong Jinhuan Medical Products Co., Ltd., Shanghai, China); Recombinant Human Type III Collagen (Jiangsu Trautec Medical Technology Co., Ltd., Changzhou, China); DH5α glycerol bacteria, CD31 antibody, and TGF-β3 antibody (Beyotime Biotechnology Co., Ltd., Shanghai, China); TGF-β1 antibody (Servicebio Biotechnology Co., Ltd., Wuhan, China); Fish sperm DNA and PEI_25K_ (Sigma-Aldrich Co., Ltd., St. Louis, MO, USA); ethidium bromide (Beijing Boaosen Biotechnology Co., Ltd., Beijing, China); Lipo8000 and the BCA protein assay kit (Beyotime Biotechnology Co., Ltd., Shanghai, China); DMEM culture medium and fetal bovine serum (FBS) (Adamas Reagent Co., Ltd., Shanghai, China); the 3T3 and hESCs cell line (ATCC, Manassas, VA, USA); 3-(4,5-Dimethylthiazol-2-yl)-2,5-Diphenyltetrazolium Bromide (MTT, purity > 98%) (BioFroxx, Einhausen, Germany); DMSO (Aladdin Reagent Shanghai Co., Ltd., Shanghai, China). All the above reagents were used as purchased, with reference to the corresponding instruction manuals.

### 2.2. Surface Morphology of Sutures Loaded with DNA

Using an analytical balance, an appropriate amount of Fish sperm DNA powder was weighed and dissolved in deionized water, followed by vortex mixing to ensure homogeneity. The DNA solution was then added to the Lipo8000 solution at a ratio of 1 μg DNA to 1.6 μL Lipo8000 solution, and gently pipetted to mix thoroughly. This resulted in the preparation of a Lipo8000/DNA solution with a concentration of 10 μg/mL. The sutures were divided into two groups: one group remained untreated, while the other group was immersed in the Lipo8000/DNA solution for 20 min. After immersion, the sutures were allowed to dry in a fume hood. Subsequently, scanning electron microscopy (SEM) (Regulus 8100, Hitachi High-Technologies Corporation, Shanghai, China) was utilized to observe the surface morphology of both groups of sutures.

### 2.3. Characterization of Sutures Loaded with EB-DNA

Ethidium bromide, as a small molecule organic dye compound, has been confirmed to be capable of intercalating into the base pairs of DNA molecules [38]. In our experiments, ethidium bromide (EB) was combined with the DNA solution at a mass ratio of 10:1. In our article, it will be subsequently referred to as EB-DNA. The sutures were categorized into three groups, with one serving as the control. In one of the experimental groups, the sutures were directly immersed in the EB-DNA solution. In the other experimental group, the sutures were first immersed in 1 mg/mL RhCol III solution for 30 min prior to immersion in the EB-DNA solution. After both groups of sutures had been immersed in the EB-DNA solution and subsequently dried in a fume hood, an inverted fluorescence microscope (Nikon Ti2, Nikon Corporation, Shanghai, China) was employed to observe the attachment of the EB-DNA onto the sutures.

### 2.4. Particle Size and Zeta Potential of DNA

To ensure that the DNA was suitable for loading onto sutures in terms of particle size and zeta potential, and was consistent with the particulate matter observed on the sutures in the morphological characterization, we selected two commonly used non-viral vectors for gene loading, namely PEI_25K_ and Lipo8000. The PEI_25K_/DNA solution was prepared at a mass ratio of 1:1. Specifically, 1 mL of a 10 μg/mL PEI_25K_ solution was taken, and 1 mL of a 10 μg/mL DNA solution was added under vortexing. The mixture was then thoroughly mixed and awaited testing. In addition, a Lipo8000/DNA solution was prepared at a ratio of 1.6 μL of Lipo8000 per 1 μg of DNA. Specifically, 1 mL of a solution containing 16 μL of Lipo8000 was taken, and 1 mL of a 10 μg/mL DNA solution was slowly added. The mixture was gently mixed. A Malvern particle size analyzer (Malvern Zetasizer Nano ZS90, Malvern Panalytical Ltd., Shanghai, China) was used to measure the particle size, size distribution, and zeta potential of both the PEI_25K_/DNA solution and the Lipo8000/DNA solution.

### 2.5. DNA Loading Capacity of Sutures

Using ethidium bromide (EB) to label DNA, an EB-DNA solution was prepared at a mass ratio of 10:1. Sutures were cut into 2 cm segments and immersed in a 1 μg/mL EB-DNA solution. A fluorescence spectrophotometer equipped (Edinburgh FS5, Edinburgh Instruments., Shanghai, China) with a 50 μL ultra-micro fluorescence cuvette was utilized to detect the difference in EB-DNA loading capacity between sutures loaded with and without RhCol III.

### 2.6. In Vitro Release of DNA from Sutures

Segments of sutures that had adsorbed a 1 μg/mL EB-DNA solution were placed into 1.5 mL centrifuge tubes, and 60 μL of solution was added. At specific time points, 50 μL of the release solution was removed and replaced with an equal volume of PBS. The concentration of the release solution was determined using a fluorescence spectrophotometer, and the cumulative release rate from the sutures was calculated.

### 2.7. Cellular Transfection Experiments with Medicine-Loaded Sutures

To evaluate the expression capacity of sutures after releasing substances, we first used 293T cells, which are commonly used in transfection experiments, for validation. 293T cells were seeded into a 24-well plate at a density of 100,000 cells per well and incubated in a 37 °C incubator for 16–18 h. Subsequently, the sterile sutures were taken out and cut into 1 cm long segments. A negative control group (without transfection experiments) and four experimental groups were established in total. Each group had at least three wells, and each well contained 5 cm of sutures. Three of the experimental groups were soaked in recombinant human type III collagen (RhCol III) solutions at concentrations of 0.1 mg/mL, 0.5 mg/mL, and 1 mg/mL, respectively, for 30 min (the RhCol III solutions were diluted with DMEM medium without fetal bovine serum). After soaking, the sutures were air-dried in a biosafety cabinet for subsequent use. The sutures in the fourth group were not treated with RhCol III (this group served as the positive control group). When the cell confluence reached 80%, preparations for the transfection experiment were initiated. The medium in the well plate was discarded, and 500 μL of serum-free DMEM medium was added to each well. The Lipo8000/TGF-β3 solution was prepared at a ratio of 1.6 μL of Lipo8000 solution per 1 μg of TGF-β3 plasmid, dispensed into multiple 1.5 mL centrifuge tubes, and labeled according to the groups. Using ophthalmic forceps, the pre-treated suture segments from different groups were placed into the corresponding Lipo8000/TGF-β3 solutions. Each group had at least three wells. The sutures to be added to each well were infiltrated with the Lipo8000/TGF-β3 solution containing 1 μg of TGF-β3 plasmid for 20 min (for the positive control group, the Lipo8000/TGF-β3 solution containing 1 μg of TGF-β3 plasmid was directly added to each well). Finally, the sutures were taken out with ophthalmic forceps and placed into the corresponding cell wells. After incubating the well plate in a 37 °C cell incubator for 4–6 h, the serum-free DMEM medium in the well plate was replaced with DMEM medium containing 10% fetal bovine serum. After 48 h, photographs were taken under an inverted fluorescence microscope, and the transfection efficiency was quantitatively analyzed using a flow cytometer. The same experimental procedures were applied when conducting experiments on human endometrial stromal cells (hESCs).

### 2.8. Western Blot

hESCs were plated in a 6-well plate at a density of 500,000 cells per well. After 18–24 h, the cells reached confluence and were co-incubated with sutures loaded with RhCol III and TGF-β3. The experiment was divided into three groups: a blank control group, a RhCol III +Lipo8000 group, and a RhCol III +Lipo8000/TGF-β3 group. After 48 h of incubation, the samples were retrieved, and the cells were washed three times with PBS. The cells were then detached using a cell scraper and collected in 1.5 mL centrifuge tubes. The cells were centrifuged at 3000 rpm for 5 min at 4 °C. After removing the supernatant, 250 μL of RIPA lysis buffer (containing protease and phosphatase inhibitors) was added, and the mixture was lysed on ice for 30 min. Subsequently, the mixture was centrifuged at 12,000 rpm for 15 min at 4 °C. The supernatant was collected, and the protein concentration was determined using a BCA protein assay kit. Equal amounts of protein were subjected to electrophoresis on a 15% SDS-PAGE gel and then transferred to a PVDF (polyvinylidene fluoride) membrane. After blocking the membrane with 5% skimmed milk powder at room temperature for 1 h, it was incubated with a TGF-β3 antibody overnight at 4 °C. Following three washes with TBST, the membrane was incubated with horseradish peroxidase-conjugated goat anti-rabbit IgG at room temperature for 1 h. A β-actin antibody was used as a loading control. A quantitative analysis was performed using a Tanon 5200 Multi (Tanon, Shanghai, China), and the relative expression levels were calculated with respect to the corresponding internal control β-actin.

### 2.9. Cell Proliferation Assay for RhCol III

The cell proliferation assay employed the MTT (3-(4,5-Dimethylthiazol-2-yl)-2,5-Diphenyltetrazolium Bromide) method to evaluate the proliferative effect of recombinant human type III collagen (RhCol III) on cells. First, the RhCol III samples were gradient-diluted using serum-free DMEM medium to obtain seven concentrations: 0.01, 0.02, 0.05, 0.1, 0.2, 0.5, and 1 mg/mL. Meanwhile, non-cytotoxic bovine serum albumin (BSA) was selected as the control group, with the same dilution method and concentrations as those of RhCol III mentioned above. In addition, serum-free DMEM medium was used as the negative control. Human endometrial stromal cells (hESCs) and fibroblasts (3T3) in the logarithmic growth phase were seeded into 96-well plates at a density of 5000 cells per well. After 24 h of incubation, the medium was discarded, and 100 μL of RhCol III samples at the corresponding concentrations were added to each well. Subsequently, the plates were incubated in an incubator at 37 ± 1 °C with 5% CO_2_ for 24 h. Then, 20 μL of MTT was added to each well and incubated for another 4 h. After removing the MTT dye, 150 μL of dimethyl sulfoxide (DMSO) was added. The plates were then placed on a shaker and shaken for 15 min. An analysis was performed using a microplate reader at a wavelength of 570 nm. The formula for calculating cell viability is as follows:

$$\mathrm{Cell}\;\mathrm{viability}\;(\%)=\frac{\mathrm{OD}570(\mathrm{sample})}{\mathrm{OD}570(\mathrm{control})}\;\times 100$$

### 2.10. Cell Migration Assay for RhCol III

In the cell migration assay, black marking pens were used to draw three equally spaced horizontal lines at the bottom of each well in a 6-well plate. Fibroblasts (3T3) were seeded at a concentration of 500,000 cells per well and incubated for 24 h until confluence was achieved. Afterward, a sterile pipette tip and ruler were employed to create vertical scratches at the bottom of each well. The complete culture medium in the wells was then discarded, and the wells were washed three times with PBS. Subsequently, 2 mL of six different concentrations of RhCol III solutions (0, 0.01, 0.05, 0.1, 0.5, and 1 mg/mL), pre-diluted in culture medium, were added to the respective wells. A consistent field of view was identified for each group, and photographs were taken at 0, 12, 24, and 36 h to observe cell migration. ImageJ software (v1.53t) was utilized for quantitative analysis to calculate the cell migration rates at different time points for each group. The same experimental procedure was applied to human endometrial stromal cells (hESCs).

### 2.11. Construction of a Rat Model for Uterine Scar

This study obtained sexually mature female Sprague Dawley rats, aged 8 weeks and weighing between 200 and 220 g, from the Shanghai Experimental Animal Center. The rats were allowed to acclimate for one week. The estrus cycle was determined using vaginal smear cytology. Rats in normal estrus were selected for modeling, and a uterine scar model was established using intrauterine scratching techniques, including a longitudinal uterine incision followed by suturing. Briefly, the rats in estrus were anesthetized, and their abdominal cavities were opened to expose the Y-shaped uterus. A 2 mm transverse incision was made 0.5 cm away from the cervix of each uterine horn. A homemade scraping spoon was used to scrape the endometrium until the uterine cavity wall became rough and bled, and the uterine wall became thin and translucent. A 1 cm longitudinal incision was then made upwards from the transverse incision using surgical scissors. The incision was sutured using sutures under different treatment conditions. After completion of the suturing, the uterus was returned to the abdominal cavity. The abdominal cavity was then rinsed with saline, and the abdomen was sutured in layers. All animal experiments adhered to the Guidelines for the Care and Use of Laboratory Animals at Changzhou University and were approved by the Animal Ethics Committee of Changzhou University (Ethical code: 20230310056; Date: 3 March 2023).

### 2.12. In Vivo Experiments of Medicine-Loaded Sutures

Rats were randomly assigned to five distinct groups: (i) the sham group, which underwent a 2 mm transverse incision on the uterus followed by suturing without any curettage; (ii) the scar model group, in which a model of uterine scar was established and sutured using untreated sutures; (iii) the RhCol III group, where a uterine scar model was created, and the sutures were soaked in RhCol III solution for 30 min followed by adsorption of Lipo8000/Fish sperm DNA solution for 20 min, then air-dried before suturing; (iv) the TGF-β3 group, where a uterine scar model was established, and the sutures were soaked in the Lipo8000/TGF-β3 solution for 20 min, then air-dried before suturing; (v) the RhCol III+TGF-β3 group, in which a uterine scar model was created, and the sutures soaked in RhCol III solution for 30 min were further used to adsorb the Lipo8000/TGF-β3 solution for 20 min, followed by air-drying before suturing. Two weeks after the surgery, the rats were euthanized and the uterine tissues were collected.

### 2.13. Immunohistochemical Staining

Uterine tissues were fixed using 4% paraformaldehyde tissue fixative, followed by paraffin embedding, sectioning, dewaxing, and hydration at room temperature. Immunohistochemical staining was conducted using antibodies specific for CD31, TGF-β3, and TGF-β1. The pathological images were processed with image browsing software (CaseViewer v2.3). Subsequently, four random fields of view at different magnifications were selected, and the corresponding proportions of positive staining areas were analyzed using the Image J software tool.

### 2.14. Evaluation of Antifibrotic Effects

Uterine tissues were fixed with 4% paraformaldehyde tissue fixative. Subsequently, they were embedded in paraffin, sectioned, dewaxed, and hydrated at room temperature. The samples underwent staining with both Masson’s trichrome and Sirius red. Pathological images were analyzed for Masson’s trichrome staining using image browsing software (CaseViewer). Four random fields of view were selected at various magnifications. Finally, the Image J software tool was employed to process the data and assess the fibrosis rate. The sections stained with Sirius red were photographed under polarized light to observe the distribution of Collagen I and RhCol III, providing a comprehensive evaluation of the antifibrotic efficacy.

### 2.15. Statistical Analysis

All experimental data are presented as mean ± standard deviation. Statistical significance for multiple comparisons was determined using one-way analysis of variance. The quantification of all dyed pictures was performed using Image J, and the statistical analysis was performed using GraphPad Prism 8 or Origin 2022. *p* < 0.05 was considered statistically significant.

## 3. Results

### 3.1. Feasibility of Medicine Loading on Sutures and Related Characterizations

**Figure 2:**
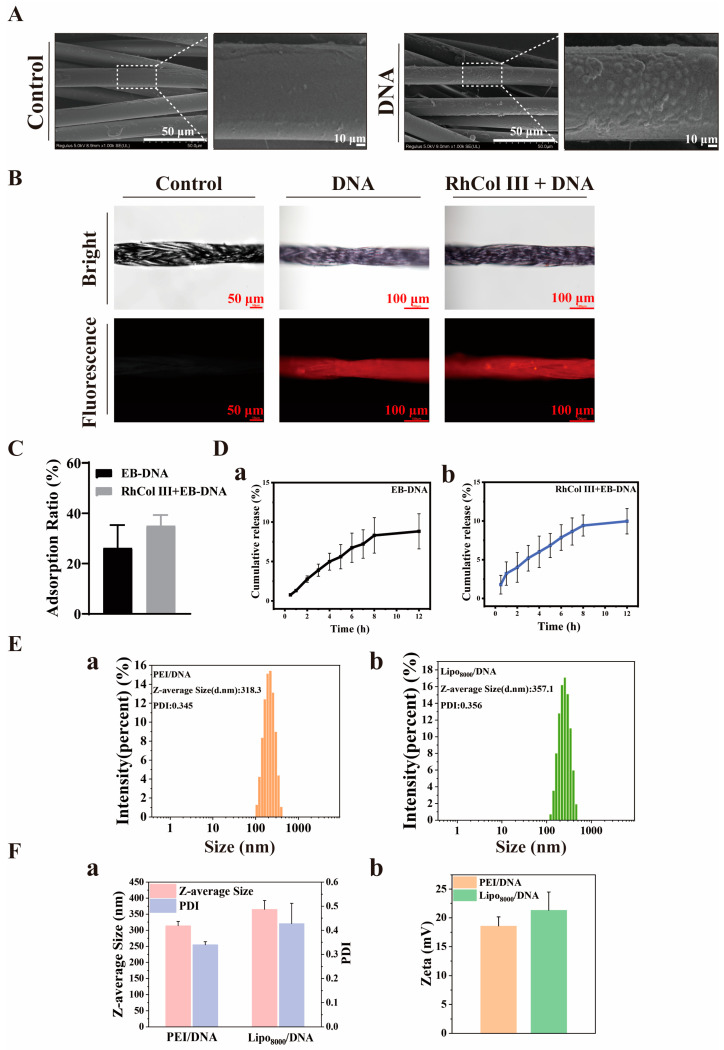
Feasibility and related characterization of medicine loading on sutures. (**A**) Scanning Electron Microscope (SEM) images of sutures before and after DNA adsorption. Scale bar: 50 μm; 10 μm. (**B**) Inverted fluorescence microscope images of EB-DNA adsorbed onto sutures. Scale bar: 50 μm; 100 μm. (**C**) Changes in DNA adsorption capacity of sutures before and after the adsorption of RhCol III. (**D**) Cumulative release rate of DNA within 12 h from sutures before (**a**) and after (**b**) collagen adsorption. (**E**) Particle size distribution of DNA loaded onto different carriers, PEI_25K_ (**a**) and Lipo8000 (**b**). (**F**) (**a**) Particle size and (**b**) zeta potential. Each value represents the mean ± SD.

To ascertain the changes in absorbable sutures before and after immersion and the adsorption of DNA, Scanning Electron Microscopy (SEM) was employed to observe the surface morphology of the sutures before and after DNA loading. It was observed that following immersion in DNA solution, punctate or spherical attachments emerged on the surface of the sutures (Figure 2A). Based on this observation, to further confirm the successful loading of DNA onto the sutures, ethidium bromide was utilized as a fluorescent marker. After binding with DNA, the complex was uniformly mixed with Lipo8000 and then applied to the sutures. Upon the completion of the soaking process, an inverted fluorescence microscope was used to observe the sutures, revealing the presence of particulate matter on the sutures. Moreover, the DNA labeled with ethidium bromide exhibited an orange-red fluorescence (Figure 2B), which corresponded with the previous SEM results. This indicates that the PGA sutures can successfully load the target DNA through a simple adsorption process.

To evaluate the DNA loading efficacy on sutures, we labeled DNA with ethidium bromide (EB). Subsequent analysis using a fluorescence spectrophotometer revealed a peak absorption at 630 nm for the EB-DNA solution. By comparing the experimental groups to a standard curve, we determined that the adsorption capacity of sutures without prior RhCol III pretreatment was 22.18%, whereas the adsorption capacity of sutures with RhCol III pretreatment was 27.71%. This represents an increase in adsorption capacity by 5.53% (Figure 2C).

To assess the release performance of DNA from the two groups of sutures after adsorption, we measured the release solution at various time points using a fluorescence spectrophotometer. The results indicated that the cumulative release rate at 12 h for the suture group without RhCol III was 8.82%, while the cumulative release rate for the suture group with RhCol III was 9.96% (Figure 2D). This suggests that the addition of RhCol III did not adversely affect the release capacity of the sutures.

When PEI_25K_ and Lipo8000 were utilized as DNA carriers, respectively, a uniform particle size distribution of the DNA was observed (Figure 2E(a,b)). When PEI_25K_ served as the carrier, the particle size was 314 nm with a zeta potential of 18.6 mV. In contrast, when Lipo8000 was used as the carrier, the particle size was 364.7 nm with a zeta potential of 21.3 mV (Figure 2F(a,b)). These observations are consistent with the DNA particle morphologies characterized above. Additionally, the positively charged solutions facilitate subsequent binding to negatively charged cell membranes in further experiments.

### 3.2. In Vitro Experiments of Medicine-Loaded Sutures

**Figure 3:**
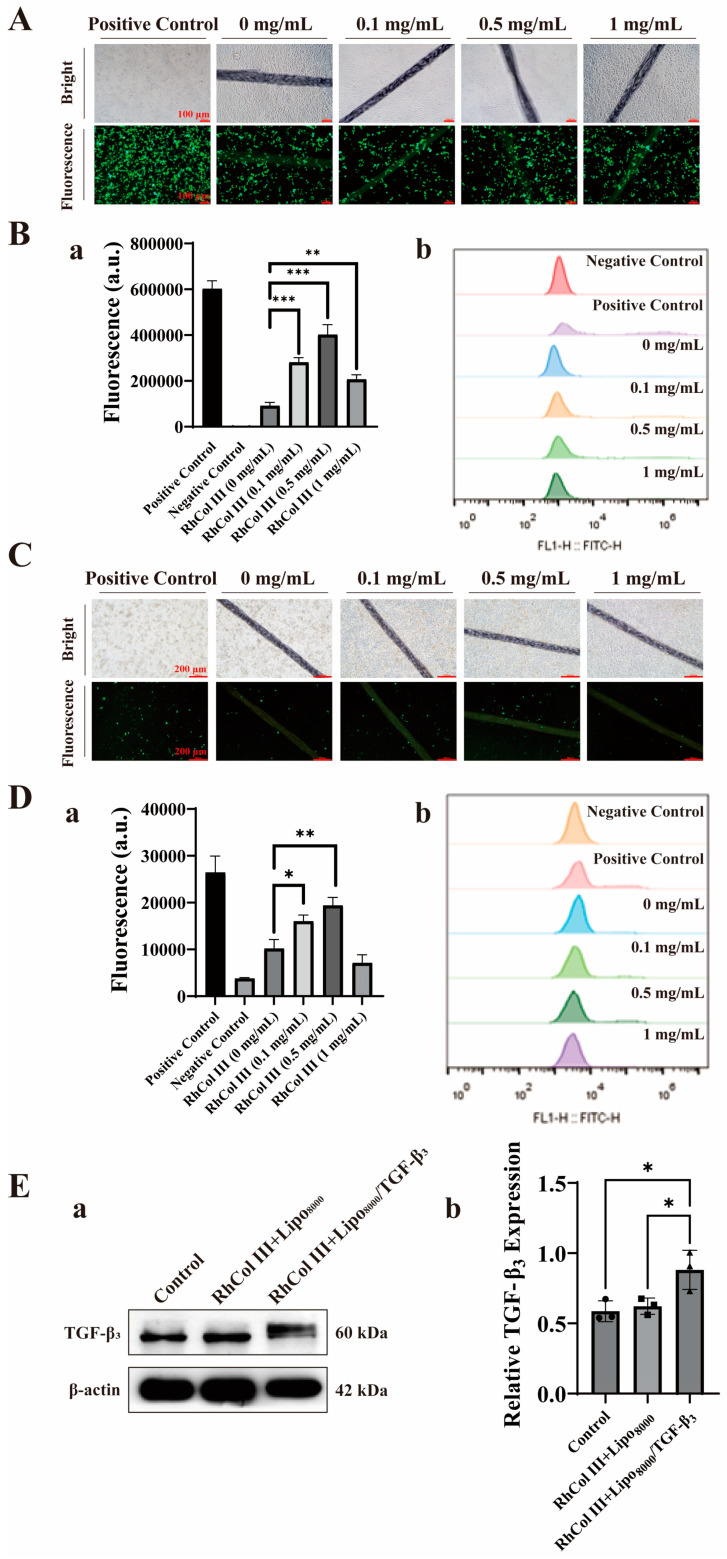
In vitro expression of medicine-loaded sutures. (**A**,**C**) Expression results of TGF-β3 in 293T cells (scale bar: 100 μm) and human endometrial stromal cells (hESCs) (scale bar: 200 μm) for sutures coated with different concentrations of RhCol III. (**B**) (**a**,**b**) Flow cytometry-based quantitative analysis of transfection efficiency in 293T cells. (**D**) (**a**,**b**) Flow cytometry-based quantitative analysis of transfection efficiency in hESCs. (**E**) (**a**,**b**) Western blot analysis of hESCs after transfection for 48 h. Each value represents the mean ± SD. (the more asterisks (*) there are, the stronger the significant difference indicates, * *p* < 0.05, ** *p* < 0.01, *** *p* < 0.001). Due to the inherent characteristics of the cells, the transfection efficiency of hESCs (human endometrial stromal cells) was significantly weaker than that of 293T cells (**C**). Quantitative analysis using flow cytometry showed that sutures treated with RhCol III at a concentration of 0.5 mg/mL demonstrated better transfection efficiency, which was consistent with the previous fluorescence quantitation results obtained with 293T cells (**D**) (**a**,**b**).

RhCol III has been well-documented for its effectiveness in promoting cellular activity [39,40]. To investigate the release activity of sutures loaded with RhCol III and the gene medicine TGF-β3, sutures treated with varying concentrations of RhCol III were soaked in the Lipo8000/TGF-β3 solution. After the co-incubation of the sutures from different groups with 293T cells for 48 h, inverted fluorescence microscopy was used to capture images, revealing strong green fluorescence. Among them, the sutures soaked in RhCol III at a concentration of 0.5 mg/mL exhibited the strongest fluorescence effect in the microscopic field of view (Figure 3A). To further determine the impact of different concentrations of RhCol III on transfection efficiency, flow cytometry was employed to quantitatively analyze the fluorescence intensity of cellular uptake capacity. The results indicated that all concentrations of RhCol III enhanced transfection efficiency, with the best effect observed at a concentration of 0.5 mg/mL (Figure 3B(a,b)), which was consistent with the previously observed effects in the microscopic field of view.

In addition, we conducted Western blot experiments to assess the expression of exogenous transforming growth factor-β3 (TGF-β3) in human endometrial stromal cells (hESCs) after its introduction. Compared with the blank control group, the Col 3+Lipo8000/TGF-β3 group exhibited a significant increase in TGF-β3 protein expression (Figure 3E(a,b)). The second group served as a control to exclude the influence of non-plasmid factors on TGF-β3 expression, aligning with our previous expectations.

**Figure 4:**
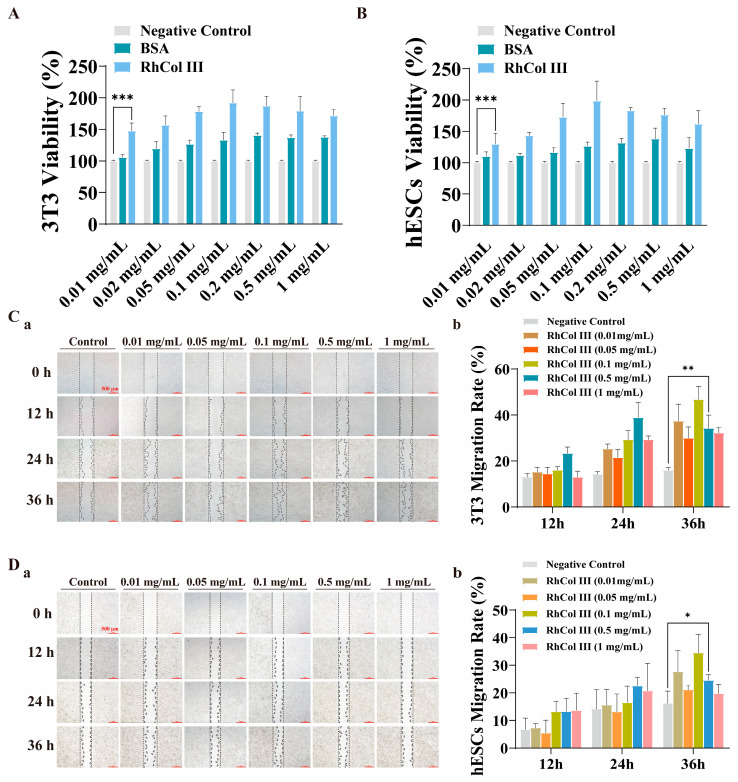
The ability of RhCol III to promote cell proliferation and migration. (**A**,**B**) The proliferative effects of various concentrations of RhCol III on 3T3 cells and hESCs. (**C**) (**a**,**b**) The migration-promoting effect of different concentrations of RhCol III on 3T3 cells at various time points (scale bar: 500 μm) and statistical analysis of cell migration rates. (**D**) (**a**,**b**) The migration-promoting effect of different concentrations of RhCol III on hESCs (scale bar: 500 μm) and statistical analysis of cell migration rates. (the more asterisks (*) there are, the stronger the significant difference indicates, * *p* < 0.05, ** *p* < 0.01, *** *p* < 0.001).

Recombinant human type III collagen (RhCol III) has been reported to exhibit excellent wound-healing properties within a certain concentration range [33,41]. Based on this finding, we co-incubated 3T3 fibroblasts with RhCol III at different concentrations. The results indicated that RhCol III significantly promoted the proliferation of 3T3 cells at all the set concentrations. Notably, the optimal proliferative effect was achieved when the concentration of RhCol III was 0.1 mg/mL (Figure 4A). To further verify the proliferative effect of RhCol III on human endometrial stromal cells (hESCs), we co-incubated hESCs with RhCol III at different concentrations under the same experimental conditions. The results showed that RhCol III also effectively promoted the proliferation of hESCs, and the best proliferative effect was observed at a concentration of 0.1 mg/mL (Figure 4B).

To evaluate the cell migration-promoting effect of RhCol III, photographs were taken of each group at 0, 12, 24, and 36 h after the addition of different concentrations of RhCol III (Figure 4C(a),D(a)). Based on these photographic results, the migration rates of the two cell types were calculated. The findings indicated that at 24 and 36 h, RhCol III significantly promoted the migration of both cell types (Figure 4C(b),D(b)). The groups treated with RhCol III concentrations of 0.1 mg/mL and 0.5 mg/mL exhibited statistically significant differences compared to the control group, which was generally consistent with the previously observed cell proliferation effects.

### 3.3. The Therapeutic Effects of Medicine—Loaded Sutures on Rats with Uterine Scars In Vivo

**Figure 5:**
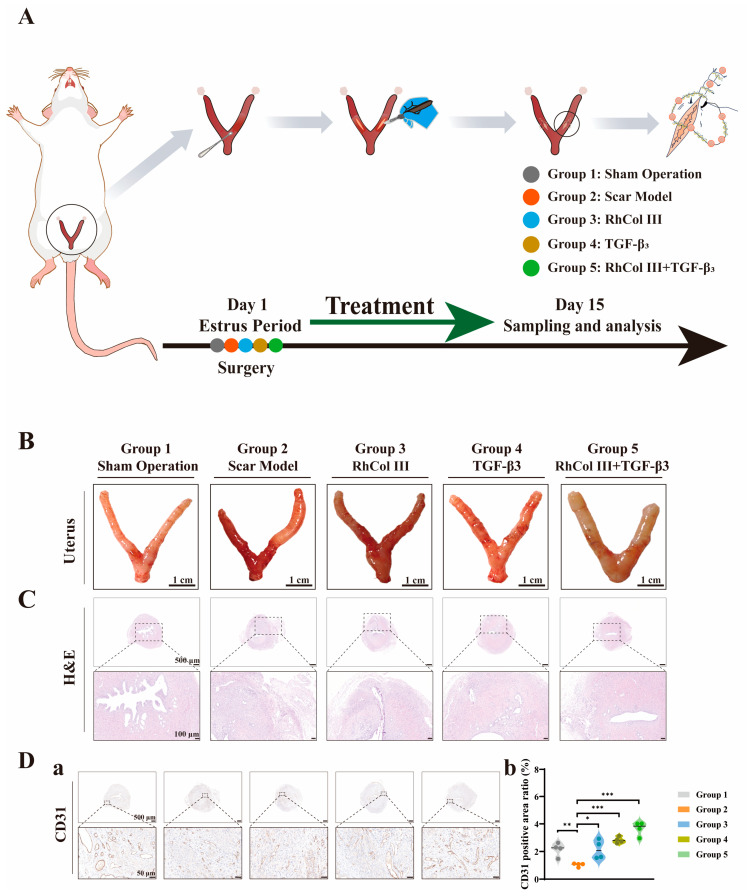
Therapeutic effects of medicine-loaded sutures on uterine scars in rats. (**A**) Schematic illustration and timeline of the surgical procedure, with different grouping arrangements. (**B**) Photographic images of uterine tissue morphology after treatment. (**C**) Corresponding hematoxylin and eosin (HE)-stained sections for different groups. Scale bars: 500 μm, 100 μm. (**D**) (**a**) Corresponding immunohistochemical staining for CD31. Scale bars: 500 μm, 50 μm. (**D**) (**b**) Quantitative analysis of CD31 protein expression levels. (the more asterisks (*) there are, the stronger the significant difference indicates, * *p* < 0.05, ** *p* < 0.01, *** *p* < 0.001).

Uterine scar is primarily a condition formed due to the inadequate healing of cesarean section wounds. Currently, there is no particularly well-established rat model for uterine scar. We have adopted the reported rat models related to cesarean scar diverticulum and intrauterine adhesions (IUAs) for comprehensive evaluation and reference [42,43,44], to assess the effectiveness of suture-mediated gene delivery systems in preventing scars and scar diverticula. To mitigate the impact of estrogens and progesterone on the uterine morphology of animals, we observed and confirmed the estrous cycle of the rats via vaginal smears [45,46]. Subsequently, surgical procedures were conducted during estrus, a phase characterized by elevated levels of estrogens and progesterone and uterine dilation, which facilitates curettage. Euthanasia and tissue sampling were performed two weeks post-surgery (Figure 5A).

Compared to the sham surgery group, the scar model group exhibited uterine cavity effusion and redness and swelling at the suture site in terms of uterine histomorphology. The RhCol III group and the TGF-β3 group displayed more pronounced redness, swelling, and blood clot accumulation compared to the RhCol III+TGF-β3 group (Figure 5B), although further analysis is required in conjunction with histological section results. An observation under hematoxylin and eosin (HE) staining revealed tissue disruption in both the serosal and muscular layers at the uterine suture site in the scar model group. In contrast, the histological sections of the TGF-β3 group and the RhCol III+TGF-β3 group appeared relatively more intact (Figure 5C).

CD31 is a crucial marker for vascular endothelial cells [47,48]. We conducted a statistical analysis of the blood vessels near the suture site, and the results showed that the positive areas in the RhCol III group, the TGF-β3 group, and the RhCol III + TGF-β3 group were significantly higher than those in the scar model group (*p* < 0.01) (Figure 5D(a,b)). In summary, the presence of either RhCol III or functional genes alone on the suture line exhibits a certain promotional effect on angiogenesis. However, the suture line carrying both RhCol III and the functional gene TGF-β3 exhibits a more significant angiogenic effect.

**Figure 6:**
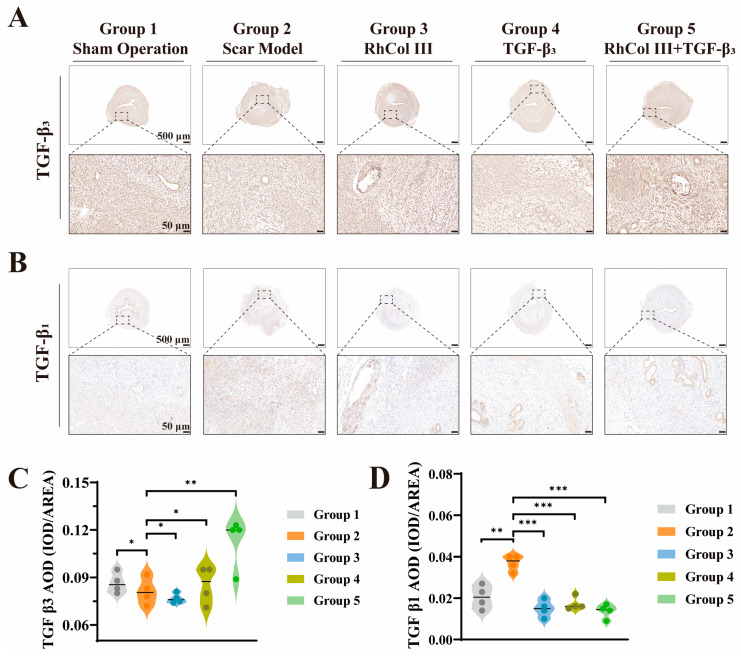
Immunohistochemical staining and expression analysis of transforming growth factor-β3 and -β1. (**A**) Corresponding immunohistochemical staining for TGF-β3. Scale bars: 500 μm, 50 μm. (**B**) Corresponding immunohistochemical staining for TGF-β1. Scale bars: 500 μm, 50 μm. (**C**) Quantitative analysis of TGF-β3 protein expression levels. (**D**) Quantitative analysis of TGF-β1 protein expression levels. (the more asterisks (*) there are, the stronger the significant difference indicates, * *p* < 0.05, ** *p* < 0.01, *** *p* < 0.001).

Furthermore, the exogenous introduction of transforming growth factor-β3 (TGF-β3) has a positive effect on reducing wound scar formation [26,30,49]. Based on this, after the exogenous introduction of TGF-β3 via sutures, immunohistochemical staining and quantitative analysis were performed on the sections. The results showed that the expression level of TGF-β3 in the model group was significantly lower than that in the sham-operated group. The damage to the uterine tissue was likely the main cause of this change. Among the three treatment groups, the expression levels of TGF-β3 in the recombinant human type III collagen (RhCol III) group and the TGF-β3 group were higher than those in the model group. However, it is worth noting that, compared with the scar model group, the expression level of TGF-β3 in the RhCol III + TGF-β3 treatment group increased by 39%, which was significantly higher than that in the other two treatment groups (Figure 6A,C). This is also consistent with our expectations. It may be due to the synergistic effect between RhCol III on the sutures and the functional genes has a positive impact on inhibiting scar formation.

In addition, we conducted immunohistochemical staining and quantitative analysis of TGF-β1 in the uterine tissue. The results indicated that the expression level of TGF-β1 in the scar model group was significantly higher than that in the control group and other treatment groups. Among the three treatment groups, the expression level of TGF-β1 in the RhCol III + TGF-β3 group was the lowest. Compared with the scar model group, it decreased by 62.8%, showing a favorable downward trend (Figure 6B,D). Therefore, we observed an upregulation of TGF-β3 expression and a downregulation of TGF-β1 expression in the treatment groups. This change will increase the TGF-β3/TGF-β1 ratio, which has a very positive effect on inhibiting tissue fibrosis and promoting the scar-free healing of the uterus [27,28,29,30].

**Figure 7:**
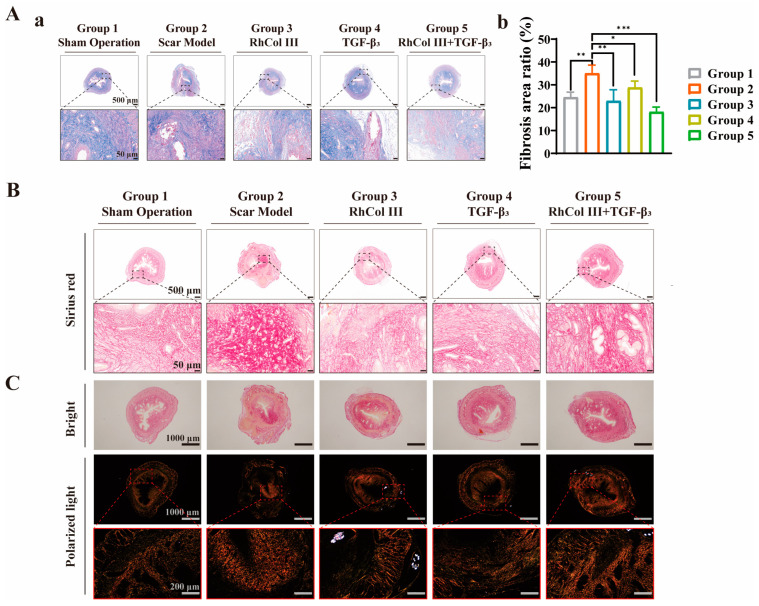
Antifibrotic effects of medicine-eluting sutures on uterine scars in rats. (**A**) (**a**) Corresponding Masson’s trichrome staining. Scale bars: 500 μm, 50 μm. (**A**) (**b**) Quantitative analysis of fibrosis. (**B**) Corresponding Sirius red staining. Scale bars: 500 μm, 50 μm. (**C**) Corresponding histological sections under polarized light. These sections indicate a reduction in the expression of Collagen I at the uterine suture site following treatment with medicine-eluting sutures. Scale bars: 1000 μm, 200 μm. (the more asterisks (*) there are, the stronger the significant difference indicates, * *p* < 0.05, ** *p* < 0.01, *** *p* < 0.001).

After Masson staining, the collagen fibers in the tissue sections appeared blue. In the model group, partial loss of the outer edge was observed during the tissue fixation stage. However, this did not affect the morphology of the endometrial and myometrial layers. Poor healing of the myometrium and pouch-like spaces resembling diverticula were clearly visible. Both the RhCol III group and the TGF-β3 group showed a reduction in blue collagen deposition to varying degrees. The uterine layer morphology of the RhCol III + TGF-β3 group was more similar to that of the intact uterine layer in the sham-operated group, with relatively less blue collagen deposition. A quantitative analysis using the ImageJ software tool revealed that compared with the scar model group, the fibrosis rate of the RhCol III + TGF-β3 group decreased by 16.8%. Moreover, compared with other groups, the RhCol III + TGF-β3 group had the smallest fibrotic area, further confirming that the synergistic effect of RhCol III and TGF-β3 had a significant antifibrotic effect (*p* < 0.01, Figure 7A(a,b)).

Under polarized light microscopy, Sirius red staining facilitates the distinction of different collagen types, with Collagen I displaying an orange-yellow hue and Collagen III appearing green. The excessive deposition of these fibrotic components may lead to a severe imbalance in the proportion of collagen types [50,51,52]. Typically, Collagen I deposition is characterized by thick, tightly arranged bundles and is predominantly found in endometrial connective tissue and scar tissue [53,54,55]. An observation under polarized light microscopy revealed that the model group exhibited more prominent orange-yellow staining near the uterine incision suture site, indicating an increase in Collagen I. This may be due to the inflammatory response triggered by uterine trauma, accompanied by fibrotic deposition. In contrast, the three different treatment groups were relatively closer to the sham-operated group, showing reduced orange-yellow staining and decreased Collagen I near the uterine wound suture site (where suture fragments appeared white under polarized light) (Figure 7B,C). Earlier literature reports have also pointed out that RhCol III itself may possess the capability to downregulate TGF-β1 [56], which similarly exerts a positive effect on promoting wound healing and counteracting stubborn connective tissue at uterine scars during subsequent treatment.

## 4. Discussion

In the initial stage of wound healing, suppressing the inflammatory response and reducing tissue fibrosis represent an ideal strategy for decreasing the incidence of scar formation. This study aims to explore the application prospects of sutures loaded with recombinant human type III collagen (RhCol III) and the gene therapeutic agent transforming growth factor-β3 (TGF-β3) in the prophylactic treatment of uterine scar diverticulum.

The research findings indicate that the gene therapeutic agent TGF-β3 can be efficiently adsorbed onto the sutures and successfully released. When the sutures are pre-loaded with RhCol III and then combined with the gene therapeutic agent, it can significantly enhance the cell transfection efficiency and cell viability in in vitro experiments, playing a positive role in promoting the scar-free healing process of uterine incisions. In vivo experiments further verify the effectiveness of the synergistic action between the gene therapeutic agent TGF-β3 and RhCol III. Specifically, when TGF-β3 or RhCol III is used alone in combination with the sutures, although it can increase the expression of some related factors, its impact on wound healing and antifibrotic activity is relatively limited. In contrast, after treatment with the RhCol III + TGF-β3 group, compared with the scar model group, the expression level of TGF-β3 has increased by 39%, the expression level of TGF-β1 has decreased by 62.8%, and the fibrosis rate has decreased by 16.8%. This set of data suggests that this combined treatment approach has significant positive implications for effectively inhibiting the abnormal proliferation of connective tissue and promoting the scar-free repair of uterine wounds.

Previous studies have mostly focused on singular treatment approaches. [17,24,33,42], such as simply using medicine therapy or relying on certain biomaterials for adjuvant treatment, etc., to achieve the goals of suppressing the inflammatory response and reducing tissue fibrosis in the initial stage of wound healing. In contrast, this study innovatively combines two components, RhCol III and TGF-β3, and uses sutures as a carrier to successfully achieve their synergistic anti-scar effect. The experimental results clearly demonstrate that when TGF-β3 or RhCol III is used alone in combination with the sutures, their effects on wound healing and antifibrotic activity are rather limited, while the combined use of the two shows remarkable results. This forms a sharp contrast with many studies that only focus on a single component.

## 5. Conclusions

This study innovatively presents a brand-new method for treating uterine scars. Skillfully, it makes use of the indispensable suture material in the surgical procedure. The recombinant human type III collagen (RhCol III) and the gene medicine transforming growth factor-β3 (TGF-β3) are directly integrated into the sutures, ensuring that after the suturing operation is completed, the medicine can precisely and fully contact the wound site.

In vitro experiments have proven that RhCol III and the plasmid can be successfully loaded onto the sutures and smoothly achieve release and expression. The results of in vivo experiments indicate that, compared with the scar model group, RhCol III and the gene medicine act synergistically, significantly increasing the TGF-β3/TGF-β1 ratio, effectively reducing the tissue fibrosis rate and the deposition of type I collagen. This achievement has opened up a completely new approach for effectively promoting the scar-free repair of uterine wounds and reducing the incidence of uterine scars.

However, although the remarkable effect of the synergistic anti-uterine scar action of RhCol III and TGF-β3 has been verified through the mediation of sutures, the specific molecular mechanisms and signaling pathways have not been thoroughly studied yet. In the future, advanced molecular biology techniques such as gene editing and proteomics can be utilized to more comprehensively and deeply analyze the underlying mechanisms of their synergistic action, thus laying a solid theoretical foundation for the further optimization of research plans.

## Data Availability

The original contributions presented in the study are included in the article, further inquiries can be directed to the corresponding authors.

## References

[1] Glenn T.L., Han E. (2021). Cesarean scar defect: Far from understood. Fertil. Steril..

[2] Betran A.P., Ye J.F., Moller A.B., Souza J.P., Zhang J. (2021). Trends and projections of caesarean section rates: Global and regional estimates. BMJ Glob. Health.

[3] Hu L.Q., Flood P., Li Y.P., Tao W.K., Zhao P.S., Xia Y., Pian-Smith M.C., Stellaccio F.S., Ouanes J.P.P., Hu F.L. (2016). No Pain Labor & Delivery: A Global Health Initiative’s Impact on Clinical Outcomes in China. Anesth. Analg..

[4] Yin S.H., Chen L., Zhou Y.B., Yuan P.B., Guo X.Y., Lu J., Ge L., Shi H.F., Wang X.X., Li L.Y. (2023). Evaluation of Cesarean Rates for Term, Singleton, Live Vertex Deliveries in China in 2020 Among Women With No Prior Cesarean Delivery. JAMA Netw. Open.

[5] Donnez O. (2020). Cesarean scar defects: Management of an iatrogenic pathology whose prevalence has dramatically increased. Fertil. Steril..

[6] Liu D.M., Yang M., Wu Q.Q. (2018). Application of ultrasonography in the diagnosis and treatment of cesarean Check for scar pregnancy. Clin. Chim. Acta.

[7] Florio P., Filippeschi M., Moncini I., Marra E., Franchini M., Gubbini G. (2012). Hysteroscopic treatment of the cesarean-induced isthmocele in restoring infertility. Curr. Opin. Obstet. Gynecol..

[8] Guan Z.K., Liu J., Bardawil E., Guan X.M. (2020). Surgical Management of Cesarean Scar Defect: The Hysteroscopic-Assisted Robotic Single-Site Technique. J. Minim. Invasive Gynecol..

[9] Sun Q., Tang L., Zhang D. (2023). Molecular mechanisms of uterine incision healing and scar formation. Eur. J. Med. Res..

[10] Tsuji S., Nobuta Y., Hanada T., Takebayashi A., Inatomi A., Takahashi A., Amano T., Murakami T. (2023). Prevalence, definition, and etiology of cesarean scar defect and treatment of cesarean scar disorder: A narrative review. Reprod. Med. Biol..

[11] Mancuso A.C., Maetzold E., Kowalski J., Van Voorhis B. (2021). Surgical repair of a cesarean scar defect using a vaginal approach. Fertil. Steril..

[12] Timor-Tritsch I.E. (2021). Cesarean scar pregnancy: A therapeutic dilemma. Ultrasound Obstet. Gynecol..

[13] Qian Z.D., Huang L.L., Zhu X.M. (2015). Curettage or operative hysteroscopy in the treatment of cesarean scar pregnancy. Arch. Gynecol. Obstet..

[14] Chiang Y.C., Tu Y.A., Yang J.H., Lin S.Y., Lee C.N., Shih J.C. (2017). Risk factors associated with failure of treatment for cesarean scar pregnancy. Int. J. Gynecol. Obstet..

[15] Maheux-Lacroix S., Li F.N., Bujold E., Nesbitt-Hawes E., Deans R., Abbott J. (2017). Cesarean Scar Pregnancies: A Systematic Review of Treatment Options. J. Minim. Invasive Gynecol..

[16] Egorova A., Maretina M., Krylova I., Kiselev A. (2024). Polycondensed Peptide-Based Polymers for Targeted Delivery of Anti-Angiogenic siRNA to Treat Endometriosis. Int. J. Mol. Sci..

[17] David A.L. (2017). Maternal uterine artery VEGF gene therapy for treatment of intrauterine growth restriction. Placenta.

[18] Nair S., Curiel D.T., Rajaratnam V., Thota C., Al-Hendy A. (2013). Targeting adenoviral vectors for enhanced gene therapy of uterine leiomyomas. Hum. Reprod..

[19] Kauffman R.P. (2016). Treatment of cesarean scar pregnancy: Another chapter in the fertility preservation saga. Fertil. Steril..

[20] Pakyari M., Farrokhi A., Maharlooei M.K., Ghahary A. (2013). Critical Role of Transforming Growth Factor Beta in Different Phases of Wound Healing. Adv. Wound Care.

[21] Petersen K.B., Hoffmann E., Larsen C.R., Nielsen H.S. (2016). Cesarean scar pregnancy: A systematic review of treatment studies. Fertil. Steril..

[22] Yang M.J., Cao L.L., Yan J.Y., Tang Y.B., Cao N.Y., Huang L.L. (2023). Risk factors associated with the failure of initial treatment for cesarean scar pregnancy. Int. J. Gynecol. Obstet..

[23] Ferguson M.W.J., O’Kane S. (2004). Scar-free healing: From embryonic mechanisms to adult therapeutic intervention. Philos. Trans. R. Soc. B-Biol. Sci..

[24] Sun Q., Zhang D., Ai Q.Y., Yue Y., Wang H.J., Tang L., Yi X.L., Wang S.Y., Zheng Y. (2024). Human umbilical cord mesenchymal stem cells improve uterine incision healing after cesarean delivery in rats by modulating the TGF-β/Smad signaling pathway. Arch. Gynecol. Obstet..

[25] Amjadian S., Moradi S., Mohammadi P. (2022). The Emerging Therapeutic Targets for Scar Management: Genetic and Epigenetic Landscapes. Ski. Pharmacol. Physiol..

[26] Finnson K.W., McLean S., Di Guglielmo G.M., Philip A. (2013). Dynamics of Transforming Growth Factor Beta Signaling in Wound Healing and Scarring. Adv. Wound Care.

[27] Hu M.S., Maan Z.N., Wu J.C., Rennert R.C., Hong W.X., Lai T.S., Cheung A.T.M., Walmsley G.G., Chung M.T., McArdle A. (2014). Tissue Engineering and Regenerative Repair in Wound Healing. Ann. Biomed. Eng..

[28] Sun G.M. (2017). Pro-Regenerative Hydrogel Restores Scarless Skin during Cutaneous Wound Healing. Adv. Healthc. Mater..

[29] Guo Q.Q., Liu Z., Zheng J.J., Zhao H.P., Li C.Y. (2021). Substances for regenerative wound healing during antler renewal stimulated scar-less restoration of rat cutaneous wounds. Cell Tissue Res..

[30] Ferguson M.W.J., Duncan J., Bond J., Bush J., Durani P., So K., Taylor L., Chantrey J., Mason T., James G. (2009). Prophylactic administration of avotermin for improvement of skin scarring: Three double-blind, placebo-controlled, phase I/II studies. Lancet.

[31] Sorushanova A., Delgado L.M., Wu Z.N., Shologu N., Kshirsagar A., Raghunath R., Mullen A.M., Bayon Y., Pandit A., Raghunath M. (2019). The Collagen Suprafamily: From Biosynthesis to Advanced Biomaterial Development. Adv. Mater..

[32] Dong Z.Q., Liu Q.Y., Han X.W., Zhang X.Y., Wang X.Y., Hu C., Li X., Liang J., Chen Y.F., Fan Y.J. (2023). Electrospun nanofibrous membranes of recombinant human collagen type III promote cutaneous wound healing. J. Mater. Chem. B.

[33] Lin-Hui L., Yuan-Yuan Z., Ming-Yu L., Xu-Dong H., Yin-Jia D., Yue Z., Yang-Hong-Hong F., Ai-Fen C., Xu-Dong Z., Zheng-Li C. (2024). Recombinant Human Collagen Type III Improves Hypertrophic Scarring by Regulating the Ratio of Type I/III Collagen. J. Burn Care Res..

[34] Volk S.W., Wang Y., Mauldin E.A., Liechty K.W., Adams S.L. (2011). Diminished Type III Collagen Promotes Myofibroblast Differentiation and Increases Scar Deposition in Cutaneous Wound Healing. Cells Tissues Organs.

[35] Kong W.S., Bao Y.L., Li W., Guan D.D., Yin Y.T., Xiao Y.Q., Zhu S.H., Sun Y., Xia Z.F. (2024). Collaborative Enhancement of Diabetic Wound Healing and Skin Regeneration by Recombinant Human Collagen Hydrogel and hADSCs. Adv. Healthc. Mater..

[36] Liu W.B., Lin H., Zhao P., Xing L.N., Li J., Wang Z.H., Ju S., Shi X.L., Liu Y.H., Deng G. (2022). A regulatory perspective on recombinant collagen-based medical devices. Bioact. Mater..

[37] Wang Y., Zhang Y., Yang Y.P., Jin M.Y., Huang S., Zhuang Z.M., Zhang T., Cao L.L., Lin X.Y., Chen J. (2024). Versatile dopamine-functionalized hyaluronic acid-recombinant human collagen hydrogel promoting diabetic wound healing via inflammation control and vascularization tissue regeneration. Bioact. Mater..

[38] Galindo-Murillo R., Cheatham T.E. (2021). Ethidium bromide interactions with DNA: An exploration of a classic DNA-ligand complex with unbiased molecular dynamics simulations. Nucleic Acids Res..

[39] Shuai Q.Z., Liang Y.X., Xu X.R., Halbiyat Z., Wang X.W., Cheng J.W., Liu J.L., Huang T.J., Peng Z.W., Wang L. (2023). Sodium alginate hydrogel integrated with type III collagen and mesenchymal stem cell to promote endometrium regeneration and fertility restoration. Int. J. Biol. Macromol..

[40] Xu L.J., Liu Y.F., Tang L.Z., Xiao H., Yang Z., Wang S.F. (2022). Preparation of Recombinant Human Collagen III Protein Hydrogels with Sustained Release of Extracellular Vesicles for Skin Wound Healing. Int. J. Mol. Sci..

[41] Zhang X.Y., Huang Y.W., Luo T., Hu C., Li H.H., Fan X.J., Wang K.F., Liang J., Chen Y.F., Fan Y.J. (2024). Advanced Wound Healing and Scar Reduction Using an Innovative Anti-ROS Polysaccharide Hydrogel with Recombinant Human Collagen Type III. Acs Appl. Mater. Interfaces.

[42] Fan Y.H., Sun J.Y., Zhang Q.W., Lai D.M. (2021). Transplantation of human amniotic epithelial cells promotes morphological and functional regeneration in a rat uterine scar model. Stem Cell Res. Ther..

[43] Hu S.T., Dai Y.Y., Xin L.B., Zheng X.W., Ye Z., Zhang S.Y., Ma L. (2024). Minimally invasive delivery of human umbilical cord-derived mesenchymal stem cells by an injectable hydrogel via Diels-Alder click reaction for the treatment of intrauterine adhesions. Acta Biomater..

[44] Zhang W.Y., He Y.X., Chu Y., Zhai Y.X., Qian S., Wang X.H., Jiang P.J., Cui P.F., Zhang Y., Wang J.H. (2024). Amorphous curcumin-based hydrogels to reduce the incidence of post-surgical intrauterine adhesions. Regen. Biomater..

[45] Ang C.J., Skokan T.D., McKinley K.L. (2023). Mechanisms of Regeneration and Fibrosis in the Endometrium. Annu. Rev. Cell Dev. Biol..

[46] Tempest N., Hill C.J., Maclean A., Marston K., Powell S.G., Al-Lamee H., Hapangama D.K. (2022). Novel microarchitecture of human endometrial glands: Implications in endometrial regeneration and pathologies. Hum. Reprod. Update.

[47] Diaz-Rodriguez S., Rasser C., Mesnier J., Chevallier P., Gallet R., Choqueux C., Even G., Sayah N., Chaubet F., Nicoletti A. (2021). Coronary stent CD31-mimetic coating favours endothelialization and reduces local inflammation and neointimal development in vivo. Eur. Heart J..

[48] Edsfeldt A., Osterlund J., Sun J., Pan M., Tengryd C., Nitulescu M., Singh P., Persson A., Nilsson J., Goncalves I. (2022). Circulating CD31 reflects endothelial activity and is associated with a lower risk for cardiovascular complications. Eur. Heart J..

[49] Chang Z., Kishimoto Y., Hasan A., Welham N.V. (2014). TGF-β3 modulates the inflammatory environment and reduces scar formation following vocal fold mucosal injury in rats. Dis. Models Mech..

[50] Geuens T., Ruiter F.A.A., Schumacher A., Morgan F.L.C., Rademakers T., Wiersma L.E., Van den Berg C.W., Rabelink T.J., Baker M.B., LaPointe V.L.S. (2021). Thiol-ene cross-linked alginate hydrogel encapsulation modulates the extracellular matrix of kidney organoids by reducing abnormal type 1a1 collagen deposition. Biomaterials.

[51] Kim T.H., Yoo J.Y., Choi K.C., Shin J.H., Leach R.E., Fazleabas A.T., Young S.L., Lessey B.A., Yoon H.G., Jeong J.W. (2019). Loss of HDAC3 results in nonreceptive endometrium and female infertility. Sci. Transl. Med..

[52] Worke L.J., Barthold J.E., Seelbinder B., Novak T., Main R.P., Harbin S.L., Neu C.P. (2017). Densification of Type I Collagen Matrices as a Model for Cardiac Fibrosis. Adv. Healthc. Mater..

[53] Griffin M.F., Borrelli M.R., Garcia J.T., Januszyk M., King M., Lerbs T., Cui L., Moore A.L., Shen A.H., Mascharak S. (2021). JUN promotes hypertrophic skin scarring via CD36 in preclinical in vitro and in vivo models. Sci. Transl. Med..

[54] Hara M., Kobayakawa K., Ohkawa Y., Kumamaru H., Yokota K., Saito T., Kijima K., Yoshizaki S., Harimaya K., Nakashima Y. (2017). Interaction of reactive astrocytes with type I collagen induces astrocytic scar formation through the integrin-N-cadherin pathway after spinal cord injury. Nat. Med..

[55] Syed F., Ahmadi E., Iqbal S.A., Singh S., McGrouther D.A., Bayat A. (2011). Fibroblasts from the growing margin of keloid scars produce higher levels of collagen I and III compared with intralesional and extralesional sites: Clinical implications for lesional site-directed therapy. Br. J. Dermatol..

[56] Hosokawa R., Nonaka K., Morifuji M., Shum L., Ohishi M. (2003). TGF-β decreases type I collagen and scarring after labioplasty. J. Dent. Res..

